# The effects of Aurora Kinase inhibition on thyroid cancer growth and sensitivity to MAPK-directed therapies

**DOI:** 10.1080/15384047.2024.2332000

**Published:** 2024-03-23

**Authors:** Hannah M. Hicks, Veronica L. Nassar, Jane Lund, Madison M. Rose, Rebecca E. Schweppe

**Affiliations:** a Division of Endocrinology, Metabolism, and Diabetes, Department of Medicine, University of Colorado School of Medicine, Aurora, CO, USA; b University of Colorado Cancer Center, University of Colorado School of Medicine, Aurora, CO, USA

**Keywords:** Aurora Kinase, MAP kinase, thyroid cancer, drug resistance, BRAF, ERK1/2

## Abstract

Thyroid cancer is one of the deadliest endocrine cancers, and its incidence has been increasing. While mutations in *BRAF* are common in thyroid cancer, advanced PTC patients currently lack therapeutic options targeting the MAPK pathway, and despite the approved combination of BRAF and MEK1/2 inhibition for *BRAF-*mutant ATC, resistance often occurs. Here, we assess growth and signaling responses to combined BRAF and MEK1/2 inhibition in a panel of *BRAF-*mutant thyroid cancer cell lines. We first showed that combined BRAF and MEK1/2 inhibition synergistically inhibits cell growth in four out of six of the ­*BRAF*-mutant thyroid cancer cell lines tested. Western blotting showed that the MAPK pathway was robustly inhibited in all cell lines. Therefore, to identify potential mechanisms of resistance, we performed RNA-sequencing in cells sensitive or resistant to MEK1/2 inhibition. In response to MEK1/2 inhibition, we identified a downregulation of Aurora Kinase B (AURKB) in sensitive but not resistant cells. We further demonstrated that combined MEK1/2 and AURKB inhibition slowed cell growth, which was phenocopied by inhibiting AURKB and ERK1/2. Finally, we show that combined AURKB and ERK1/2 inhibition induces apoptosis in *BRAF-*mutant thyroid cancer cell lines, together suggesting a potential combination therapy for *BRAF*-mutant thyroid cancer patients.

## Introduction

Thyroid cancer is the most common endocrine malignancy, and its prevalence has been increasing over the last four decades.^1^ More aggressive subtypes of thyroid cancer, including advanced papillary thyroid cancer (PTC), poorly differentiated thyroid cancer (PDTC), and anaplastic thyroid cancer (ATC), are undifferentiated and therefore radioiodine-refractory.^2–4^ Thus, new therapies are needed to treat these tumors.^5,6^

Mutations of the MAP Kinase (MAPK) pathway, primarily in BRAF-V600E are highly prevalent in thyroid cancer.^7–9^ While pharmacologic inhibition of BRAF has shown some success in melanoma with activating *BRAF* mutations,^10^ similar trials in thyroid cancer have produced mixed results.^11–14^ Major limitations of therapy with single agent BRAF inhibition include intrinsic (upfront) and acquired resistance. Acquired resistance most often occurs through downstream re-activation of the MAPK pathway through the release of negative feedback.^15–19^ This has led to the FDA approval of combined BRAF and MEK1/2 inhibition in melanoma.^20–22^ More recently, combined BRAF and MEK1/2 inhibition was approved for ATC based on a small phase II study of the BRAF inhibitor, dabrafenib (BRAFi), in combination with the MEK1/2 inhibitor, trametinib (MEKi), which showed a response rate of 69% in 16 *BRAF*-mutant ATC patients,^14^ seven of which were durable at the time of reporting. However, the observed response rate has only been 56% in ATC, and based on studies in melanoma, it is expected that resistance will develop.^23^ Of interest, advanced PTC exhibits intrinsic resistance and a similar trial in *BRAF*-mutant PTC failed to show significant benefit for a BRAFi/MEKi combination compared to BRAFi alone.^24^ Thus, new therapeutic strategies are needed to combat mechanisms of resistance.

Here, we identified an induction of Aurora kinase B (AURKB) in response to MEKi in *BRAF-*mutant thyroid cancer cells that are resistant to combined BRAF/MEK1/2 inhibition. Aurora kinases are serine/threonine protein kinases that are part of the CPC (Chromosome Passenger Complex) and play a key role in chromosome segregation during mitosis.^25^ Due to their role controlling cell division, Aurora kinases (AURKs) are often overexpressed or amplified in human cancers, including PTC and ATC.^26^ AURKs have also been shown to promote progression of thyroid cancer.^27^ Thus, we hypothesized that inhibition of AURKB would sensitize cells to MAPK-directed therapies. By targeting both cell growth and division, combined inhibition of MAPK signaling and AURKB represents an attractive therapeutic alternative for patients with advanced thyroid cancer.

## Materials and methods

### Reagents

Dabrafenib (GSK2118436, BRAFi) was purchased from SelleckChem. Trametinib (GSK1120212, MEK1/2i) was purchased from LC Laboratories. Barasertib (AURKBi) was purchased from SelleckChem. SCH772984 (ERKi-SCH) was purchased from AbMole Bioscience or SelleckChem. Drugs were dissolved in dimethyl sulfoxide (DMSO).

### Cell lines and cell culture conditions

Human thyroid cancer cell lines CUTC5 (RRID:CVCL_W916) and CUTC60 (RRID:CVCL_VM61) were generated in our laboratory.^28^ Human thyroid cancer cell lines SW1736 (RRID:CVCL_3883), BCPAP (RRID:CVCL_0153), 8505C (RRID:CVCL_1054), KTC2 (RRID:CVCL_6476) CUTC5, and CUTC60 were grown in RPMI (Invitrogen) supplemented with 5% FBS (HyClone Laboratories), the T238 cell line was grown in RPMI supplemented with 10% FBS, and the TCO1 cell line was grown in DMEM supplemented with 10% FBS. All lines were maintained at 37°C in 5% CO_2_ and not used beyond passage 20. All cell lines were validated using short tandem repeat genotyping using the Applied Biosystems Identifier kit (#4322288) or Globalfiler® System (#4476135) in the Barbara Davis Center BioResources Core Facility, Molecular Biology Unit, at the University of Colorado Anschutz Medical Campus, and routinely monitored for Mycoplasma contamination using the Lonza Mycoalert system (Lonza Bioscience), as described.^29,30^

### Cell growth and viability assays

As previously described,^31^ cells (1500/well for SW1736, BCPAP, KTC2, T238, and TCO1; 1000/well for 8505C) were plated in triplicate in 96-well plates, treated with increasing concentrations of the indicated drugs for three days, and cell viability was measured using CellTiter-Glo® 2.0 reagent (Promega) using manufacturer’s protocol. Luminescence was read using Biotek Synergy H1 plate reader and cell viability was calculated by the intensity of luminescence in relation to a solvent control treated well.

### Synergy calculations

Synergy was calculated using the CalcuSyn software, which is based upon Chou and Talalay statistics. Combination Index (CI) values < 0.3 are considered to be strongly synergistic, CI values < 0.75 are considered to be moderately synergistic, and CI values > 0.75 are considered to be mildly synergistic or less.^32,33^

### Western Blots

Western blots were performed as described previously.^31^ Cells were treated with indicated drugs and harvested in CHAPS lysis buffer (10 mM CHAPS, 50 mM Tris (pH8.0), 150 mM NaCl and 2 mM EDTA) with 1× phosphatase and protease inhibitor cocktail (Roche). Protein lysates resolved on sodium dodecyl sulfate (SDS)-polyacrylamide gels were transferred to Immobilon-FL membranes (Millipore) and incubated at 4°C overnight with the indicated antibodies diluted in 1:3 Odyssey® Blocking Buffer in TBST (LI-COR) or 5% BSA in TBST. Antibodies used in western blot experiments (unless otherwise noted, all antibodies were purchased from Cell Signaling Technology): pT308AKT/RRID:AB_331163, pS473AKT/RRID:AB_329825, AKT/RRID:AB_329827, α-tubulin/RRID:AB_2617116 (CALBIOCHEM), β-actin/RRID:AB_476744 (Sigma), pCRAF/RRID:AB_2067317, CRAF/RRID:AB_2728706, EGR1/RRID:AB_2616601, pERK1/2/RRID:AB_2315112, ERK1/2/RRID:AB_10695739, fibronectin/RRID:AB_2924220, Ki67/RRID:AB_2636984, pMEK1/2/RRID:AB_2138017, MEK1/2/RRID:AB_10695868, pS380RSK/RRID:AB_330753, RSK/RRID:AB_330803, pS235-S6/RRID:AB_916156, pS240-S6/RRID:AB_10694233, S6/RRID:AB_2238583, vinculin/RRID:AB_2728768. Blots were incubated with secondary goat anti-mouse or anti-rabbit IRDye-conjugated antibodies (LI-COR), and proteins were imaged and quantified with the Odyssey CLx imager (Image Studio Acquisition Software Version 5.2.5, LI-COR). Procedural details for each antibody are listed in Table S2.2.

### Vi-cell growth assay

CUTC5, BCPAP, TCO1, or CUTC60 cells plated in duplicate in 6-well plates then treated with vehicle, MEKi (50 nM), AURKBi (100 nM), or the combination. After 72 hrs, cells were lifted using Trypsin-EDTA (Gibco), and cell count was determined using the Vi-CELL XR Cell Viability Analyzer (Beckman Coulter).

### IncuCyte ZOOM growth assays

Cells were seeded at 1,000–2,000 cells/well in 96-well plates, allowed to adhere for 24 hrs, then treated with BRAFi (50 nM), ERKi-SCH (500 nM), AURKBi (100 nM), or the combinations thereof. Confluency was measured every 4 hrs using phase-contrast images acquired using the IncyCyte ZOOM Live-Cell Imaging system (Essen Bioscience) at the University of Colorado Cancer Center Cell Technologies Shared Resource for 76 hrs.

### IncuCyte ZOOM cleaved caspase assays

Apoptosis was measured using the IncuCyte ZOOM Live-Cell Imaging system (Essen Bioscience) at the University of Colorado Cancer Center Cell Technologies Shared Resource. Cells were seeded at 1,000–2,000 cells/well in 96-well plates, allowed to adhere for 24 hrs, and treated with indicated drug in the presence of 5 μM Caspase-3/7 Apoptosis Assay Reagent (Essen Bioscience). Fluorescent and phase-contrast images were acquired every 4 hrs for 76 hrs. Images were analyzed for apoptotic and living cell count by IncuCyte ZOOM software. To calculate area under the curve (AUC) values from the apoptotic cell count obtained from IncuCyte ZOOM, AUC analyses were performed using GraphPad Prism V9.

## Results

### Combined BRAF and MEK1/2 inhibition results in synergistic growth inhibition in BRAF-mutant thyroid cancer cell lines

In order to identify mechanisms of resistance to MAPK-directed therapies, we determined the sensitivity of a panel of BRAFV600E-mutant PTC and ATC cell lines.^29^ We have previously measured area under the dose-response curve (AUC) values for a panel of 17 *BRAF-*mutant PTC and ATC cells lines to determine their sensitivity to BRAF, MEK, or ERK inhibition.^31^

**Figure 1.**
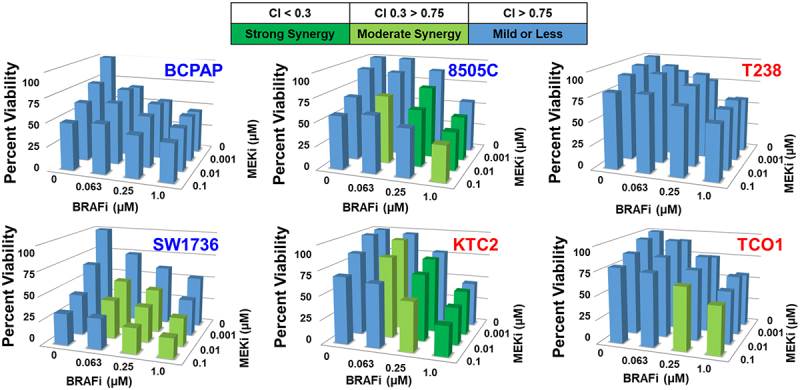
Combined BRAF and MEK1/2 inhibition results in synergistic growth inhibition in BRAFV600E-mutated cell lines. BRAFV600E-mutated thyroid cancer cell lines that are either MEKi-sensitive (BCPAP, 8505C, SW1736), indicated by the blue text, or MEKi-resistant (KTC2, T238, TCO1), indicated by the red text, were treated with indicated doses of BRAFi or MEKi for 72 hrs. Cell viability was measured using CellTiter-Glo® and synergy was determined by calculating CI values using CalcuSyn.^32^ data shown as percent growth normalized to control averaged from three individual experiments performed in triplicate. Strong synergy, Combination Index (CI) < 0.3 is shown in dark green, moderate synergy CI 0.3–0.75 is shown in light green, and blue indicates mild synergy or less (CI > 0.75). CI: combination index, BRAFi: dabrafenib, MEKi: trametinib.

Of the 17 cell lines screened previously,^31^ we chose three MEKi-sensitive (BCPAP, 8505C, SW1736) and three MEKi-resistant (T238, KTC2, TCO1) cell lines with varying growth responses to BRAFi to measure growth inhibition in response to combined BRAF and MEK1/2 inhibition. We used CellTiter-Glo® assays to measure cell viability in response to increasing doses of BRAFi and MEKi and found combined BRAF/MEK1/2 inhibition inhibits cell viability by 43–80% (Figure 1). To determine whether BRAFi and MEKi synergistically inhibit cell viability, we used the CalcuSyn Synergy calculator^32^ to calculate combination index (CI) values for each dose, where a CI value < 0.3 indicates strong synergy, a CI value < 0.75 indicates moderate synergy, and a CI value > 0.75 indicates mild to no synergy between BRAFi and MEKi. The resulting CI values show that combined BRAF and MEK1/2 inhibition synergistically inhibits cell growth in the majority of cell lines (CI value < 0.75), but not in the MEKi-resistant T238 cells that harbor a PIK3CA mutation (T238, helical domain (E542K/E545K), Figure 1). Of note, another MEKi-resistant *BRAF-*mutant cell line which also expresses a PIK3CA mutation (TCO1, kinase domain (M1043I/N1044S/H1047R)) exhibited mild to moderate synergy at most drug concentrations, indicating PIK3CA mutations themselves do not necessarily mediate resistance (Figure 1). Interestingly, the MEKi-sensitive BCPAP cell line only demonstrated mild or less synergy in response to combined BRAFi/MEKi, likely because the BCPAP cells are highly sensitive to either BRAFi or MEKi single-agent treatment, therefore, the combination confers no synergistic benefit. Raw CI values obtained using CalcuSyn are shown in Supplementary Figure S1. These data show that in a panel of *BRAF-*mutant advanced thyroid cancer cell lines sensitive or resistant to MEKi, most, but not all, exhibit moderate to strong synergistic inhibition of cell viability in response to combined BRAF/MEK1/2 inhibition.

### Combined BRAF and MEK1/2 inhibition blocks MAPK signaling and variably affects PI3K/AKT signaling

**Figure 2.**
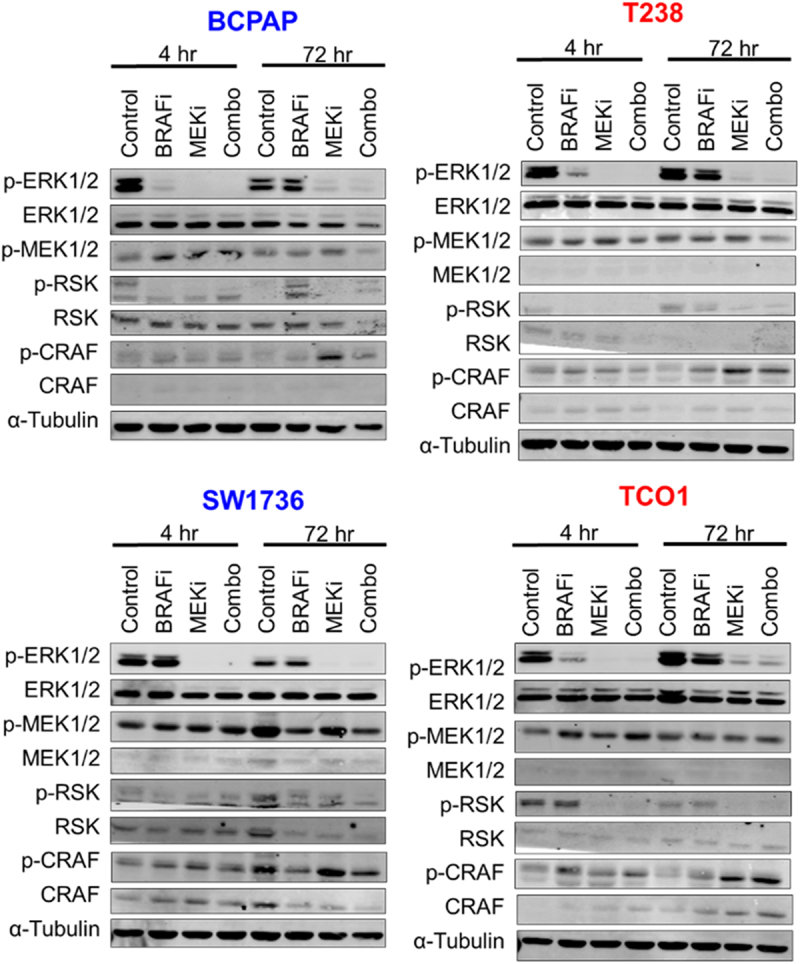
Combined BRAF and MEK1/2 inhibition blocks MAPK signaling. BRAFV600E-mutated cell lines that are sensitive (BCPAP, SW1736) or resistant (T238, TCO1) to MEK1/2 inhibition were treated with the indicated drugs for 4 hrs or 72 hrs. Indicated proteins were analyzed via western blots performed in duplicate for all cell lines except the SW1736, where *N* = 1. MEKi-sensitive cell lines are shown in blue. MEKi-resistant cells are shown in red. BRAFi: 50 nM dabrafenib, MEKi: 100 nM trametinib.

We hypothesized that cell lines which did not exhibit synergistic growth inhibition in response to combined BRAF and MEK1/2 inhibition would have less robust inhibition of the MAPK pathway. We therefore treated two MEKi-sensitive (SW1736, BCPAP) and two MEKi-resistant (T238, TCO1) cell lines with BRAFi and MEKi for 4 or 72 hrs, then measured phosphorylation of MAPK pathway nodes via western blot. We found combined BRAFi/MEKi blocks MAPK signaling, as indicated by an 83%-100% inhibition of pERK in all cell lines tested (Figure 2; Supplementary Figure S2), along with decreased pRSK. These data suggest that inadequate inhibition of the MAPK pathway is not responsible for the lack of synergy between BRAFi and MEKi observed in a subset of cell lines.

Bypass signaling via the PI3K/AKT/mTOR pathway is a common mechanism of resistance to inhibition of the MAPK pathway.^34–36^ Because we observed that the T238 cell line harboring a PIK3CA mutation does not exhibit robust inhibition of growth in response to BRAFi or MEKi, we next sought to assess activation of the PI3K/AKT/mTOR pathway. Western blot analysis of phosphorylation sites of AKT and S6, a downstream target of PI3K/AKT/mTOR signaling, in two sensitive and two resistant cell lines showed variable regulation of PI3K/AKT signaling in response to combined BRAFi/MEKi (Supplementary Figure S3). However, there was a slight trend for decreased phospho-AKT (pAKT) and pS6 levels in response to BRAF and MEK1/2 inhibition in sensitive cell lines (BCPAP, SW1736), but not in resistant cells (T238, TCO1) (Supplementary Figure S3).

### MEK1/2 inhibitor-sensitive and -resistant cell lines differentially regulate Aurora Kinases in response to MEKi

**Figure 3.**
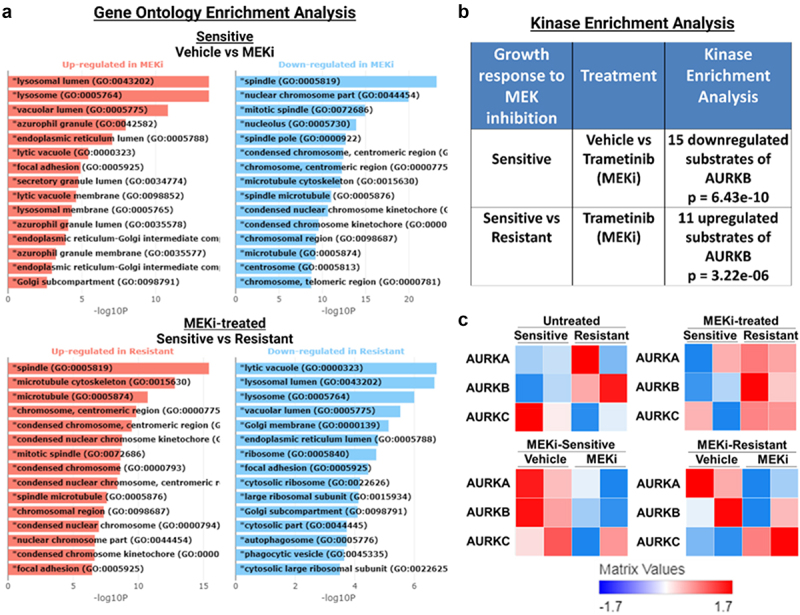
AURKB substrates are downregulated in sensitive cell lines in response to MEKi. RNA-seq was previously published,^57^ where two MEKi-sensitive (BCPAP, 8505C) and two MEKi-resistant (T238, TCO1) *BRAF*-mutant cell lines treated with vehicle or MEKi for 48 hrs. Results were analyzed using BioJupies^37^ to perform A) gene ontology enrichment analysis, B) kinase enrichment analysis, and C) generate heatmaps of AURK family protein expression. MEKi: 100 nM trametinib.

To identify potential targetable signaling nodes mediating resistance to BRAFi/MEKi treatment, we analyzed our previously published RNA-sequencing data of two MEKi-sensitive (BCPAP, 8505C) and two MEKi-resistant (T238, TCO1) cell lines treated with the MEKi, trametinib, for 48 hrs.^57^ We performed Gene Ontology (GO) Enrichment Analysis using BioJupies^37^ and found GO terms involved in cell division (spindle, nuclear chromosome part, and mitotic spindle) downregulated in response to MEKi in sensitive cell lines (BCPAP, 8505C, Figure 3a). Further, the GO terms spindle, microtubule cytoskeleton, and microtubule are upregulated in MEKi-treated resistant cell lines (T238, TCO1) compared to MEKi-treated sensitive cells (BCPAP, 8505C, Figure 3a). Interestingly, AURKA and AURKB are components of each of the top regulated GO terms (Supplementary Figure S4A). Following Kinase Enrichment Analysis (KEA), we further identified differential regulation of Aurora Kinases (Figure 3). Specifically, in response to MEKi, sensitive cells downregulated substrates of AURKB (*p* = 6.43E^−10^), while resistant cells do not (Figure 3). Further, MEKi-treated resistant cell lines exhibit an upregulation of AURKB targets compared to MEKi-treated sensitive cell lines (*p* = 3.22E^−06^). In Supplementary Figure S4B, we show the top hits from KEA and list the AURKB substrates that are up- or downregulated, as displayed in Figure 3. Of note, many of the top regulated proteins including cyclin-dependent kinase 1 (CDK1), CDK2, and polo-like kinase 1 (PLK1) are involved in cell cycle progression, similar to AURKB. Finally, in Figure 3c, we show that MEKi-resistant cell lines (T238, TCO1) have higher levels of Aurora Kinases than MEKi-sensitive cells (BCPAP, 8505C).

### Combined inhibition of MEK1/2 and AURKB slows cell growth

**Figure 4.**
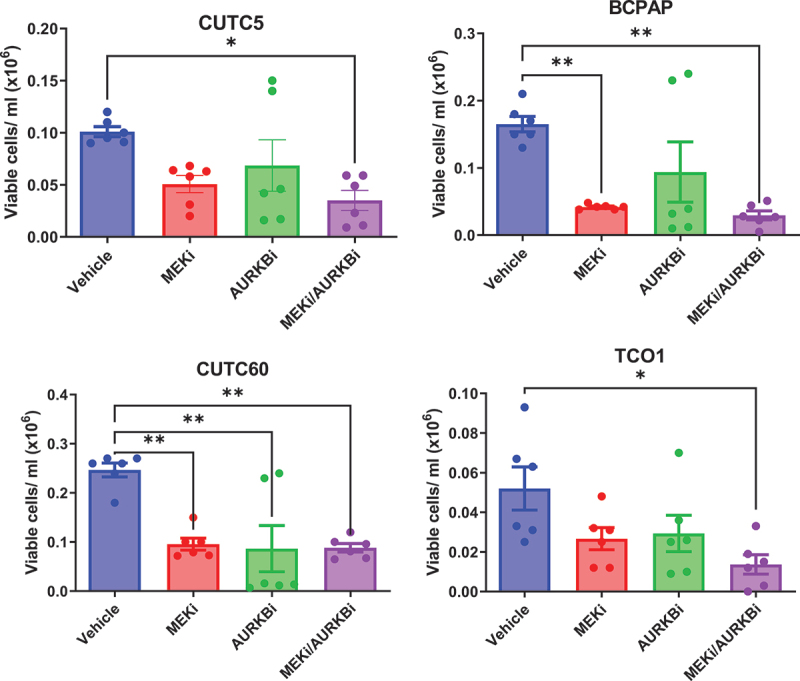
Combined MEK1/2 and AURKB inhibition slows growth of *BRAF-*mutant cell lines. *BRAF*-mutant thyroid cancer cell lines were treated with MEKi and/or AURKBi. After 72 hrs, viable cell count was determined using a vi-cell cell viability analyzer. Data shown as mean ± SEM of three experiments performed in technical duplicates. *, p-value < 0.05; **, p-value < 0.01. MEKi: 50 nM trametinib; AURKBi: 100 nM barasertib.

We hypothesized that AURKB inhibition will sensitize *BRAF-*mutant thyroid cancer cells to inhibition of the MAPK pathway. For these studies, *BRAF-*mutant thyroid cancer cells were treated with vehicle, MEKi, the AURKB inhibitor, barasertib (AURKBi), or the combination, for 72 hrs and counted using automated counting. Single-agent treatment with MEKi or AURKBi reduced cell growth in all cell lines, as did treatment with the combination. Overall, combined AURKB and MEK1/2 inhibition decreases cell growth 64–87% (Figure 4), suggesting a potential benefit of combined MEK1/2 and AURKB inhibition in *BRAF-*mutant thyroid cancer cell lines.

### Combined inhibition of the MAPK pathway and AURKB slows cell growth and increases apoptosis

**Figure 5.**
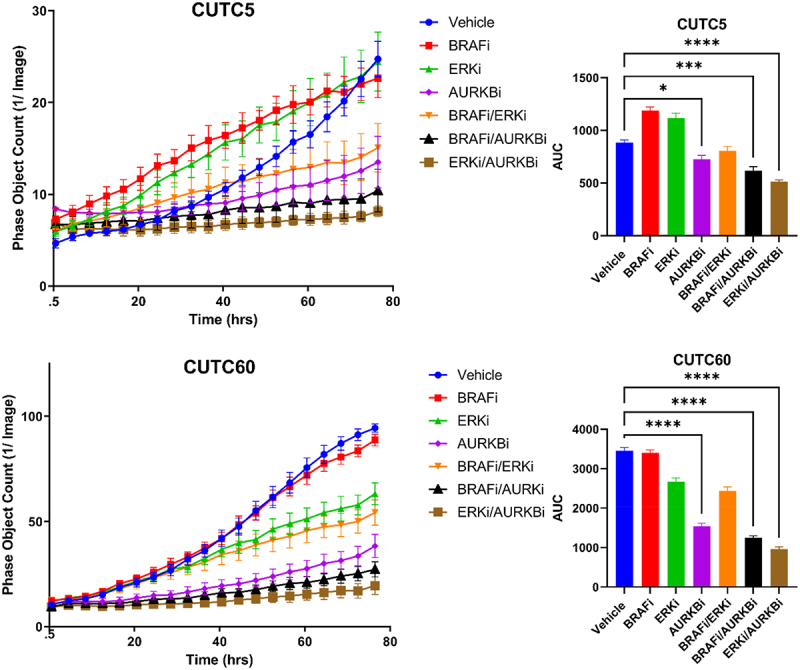
Combined ERK and AURKB inhibition slows growth of *BRAF-*mutant thyroid cancer cells. CUTC5 or CUTC60 were treated with ERKi-SCH, BRAFi and/or AURKBi. Phase object count was measured using the IncuCyte ZOOM live cell imaging system over 76 hrs. AUC was calculated using GraphPad Prism V8. Data shown as the mean ± SEM averaged from three experiments. *, p-value < 0.05; ***, p-value < 0.001; ****, p-value < 0.0001. ERKi-SCH: 500 nM SCH772984, AURKBi: 100 nM barasertib, BRAFi: 50 nM dabrafenib.

Selective ERK1/2 inhibitors have garnered interest with the rationale that targeting the most downstream node of the MAPK pathway will prevent MAPK pathway reactivation, which is the most common mechanism of resistance to MAPK-directed therapies. Inhibitors of ERK1/2 have also been shown to have activity in models of MEK1/2 inhibitor-resistance.^38,39^ Thus, we hypothesized that inhibiting ERK1/2 in combination with AURKB would prevent MAPK pathway reactivation and suppress growth to a greater extent than combined MEK1/2 and AURKB inhibition. For these studies, we treated CUTC5 and CUTC60 cell lines with BRAFi in combination with AURKBi or ERK1/2-SCH and combinations thereof. Cell growth was measured using the IncuCyte ZOOM Live Cell Imaging System to measure cell confluency over 76 hrs following treatment. Upon performing AUC analysis, we found that combined ERKi-SCH/AURKBi inhibits cell growth 42% in the CUTC5 cell line and 72% in the CUTC60 cell line (Figure 5). Together, these data indicate that compared to BRAFi combinations, combined ERKi-SCH and AURKBi inhibition most effectively inhibits cell growth.

**Figure 6.**
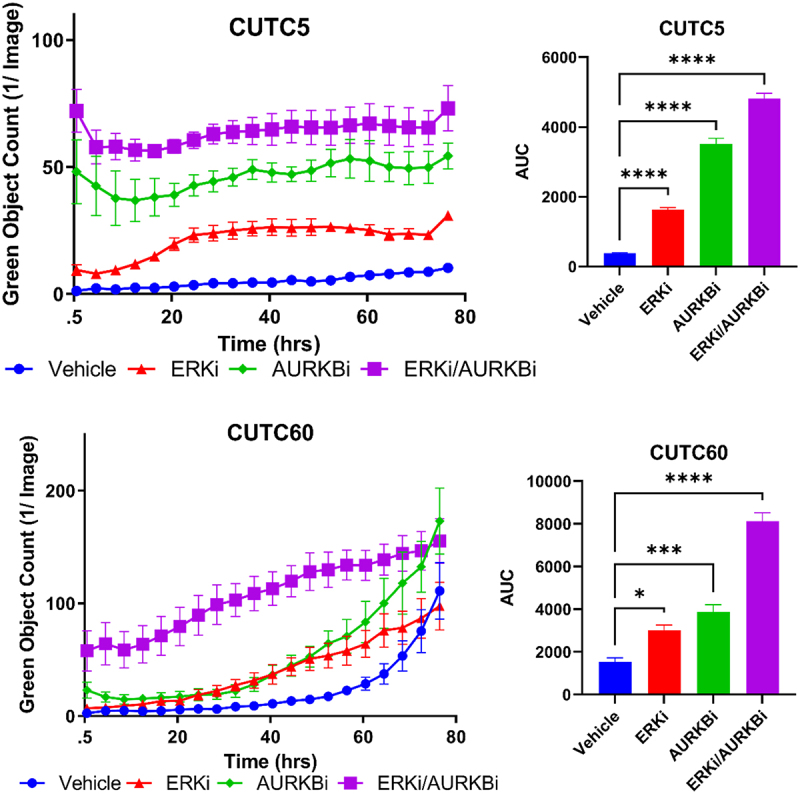
Combined ERK and AURKB inhibition increases apoptosis. *BRAF*-mutant thyroid cancer cell lines (CUTC5, CUTC60) were treated with ERKi-SCH, AURKBi or the combination, as in Fig. 5. Cleaved caspase-3/7 was measured using the IncuCyte ZOOM live cell imaging over 76 hrs. Area under the curve (AUC) was calculated using GraphPad Prism and used for statistical analysis of three experiments. *, p-value < 0.1; ***, p-value < 0.001; ****, p-values < 0.0001. ERKi-SCH: 500 nM SCH772984; AURKBi: 100 nM barasertib.

To determine how combined ERK and AURKB inhibition was slowing cell growth, we evaluated apoptosis. We measured cleaved caspase-3/7 using the IncuCyte ZOOM Live Cell Imaging System over 76 hrs following treatment with ERKi-SCH, AURKBi, or the combination in CUTC5 and CUTC60 cells. To further quantitate differences in cleaved caspase-3/7 induction between treatment groups, we performed AUC analysis. We found that combined inhibition of ERK1/2 and AURKB significantly increased apoptosis, as measured by cleaved caspase-3/7 by 5.3- to 12.7-fold at 76 hrs (Figure 6, p<.0001). Together, these data suggest that combined AURKBi and ERKi-SCH induces apoptotic cell death in *BRAF-*mutant thyroid cancer cells.

Studies have shown a correlation between levels of the transcription factor MYC and sensitivity to AURK inhibition in thyroid cancer.^40^ This is thought to be due to the ability of AURK inhibitors to disrupt AURK-MYC complexes.^40^ Specifically, AURK can form a complex with MYC to protect it from proteasomal degradation,^41,42^ whereas AURK inhibitors disrupt this complex and promote degradation of MYC.^41,42^ We mined MYC expression in a panel of seven *BRAF-*mutant cell lines using RPPA (performed by the MD Anderson Functional Proteomics Reverse Phase Protein Array Core) and graphed them in order of MYC expression (Supplementary Figure S5). Interestingly, the CUTC5 cell line had significantly lower expression of MYC than the 8505C cell line (Supplementary Figure S5A). While both cell lines appear sensitive to AURKBi, the 8505C cells are more sensitive to AURKBi than the CUTC5 cells (Supplementary Figure S5B).

## Discussion

Combined inhibition of BRAF and MEK1/2 is the only approved MAPK-directed therapeutic option for patients with *BRAF-*mutated ATC. While promising, patients often progress, making identification of new therapeutic strategies critical to patient outcome. To identify strategies to enhance MAPK-directed therapies, we tested a panel of *BRAF-*mutant PTC and ATC cell lines and showed that combined BRAFi and MEKi synergistically inhibit cell growth (Figure 1 and S1), however, some cell lines remained resistant to this combination. These resistant cells still exhibit robust inhibition of the MAPK pathway in response to combined BRAFi/MEKi (Figure 2), and while we hypothesized that upregulation in PI3K/AKT/mTOR signaling may be compensating for MAPK-pathway inhibition in resistant cells, responses were variable (Supplementary Figure S3). We therefore performed RNA-seq followed by GO term analysis and KEA to identify targetable signaling nodes that could confer resistance. Cell lines sensitive to MEKi downregulated AURKB and related substrates in response to MEK1/2 inhibition, while resistant cells did not (Figure 3). Further, AURKB expression and its substrates were upregulated in resistant cells treated with MEKi compared to sensitive cells (Figure 3). Combined inhibition of MEK1/2 or ERK1/2 in combination with AURKBi significantly decreased cell growth and combined ERK1/2 and AURKBi increased apoptosis in both sensitive and resistant cell lines (Figures 4–6). Together, these data suggest inhibition of AURKB in combination with a MAPK-directed therapy is a promising target for *BRAF-*mutant thyroid cancer.

It is interesting to note that inhibition of AURKB and ERK1/2, as opposed to other nodes of the MAPK pathway, most robustly inhibited growth in BRAFV600E mutant thyroid cancer cell lines (Figure 5). There are many potential explanations for this observation. One is that inhibiting ERK1/2 directly prevents negative feedback regulation driven by ERK1/2 phosphorylation of upstream pathway components.^43^ Consistent with this, studies in melanoma have shown that inhibition of ERK1/2 can overcome BRAF and MEKi resistance from BRAF amplification or MEK1/2 mutation^38^ as well as increased COT activity, which have been shown to occur as resistance mechanisms to MEK1/2 inhibition.^44^ Finally, inhibition of ERK1/2, as the most downstream node of the MAPK pathway, has the potential to better prevent pathway reactivation.^45^

In thyroid cancer, MYC levels were previously reported to be a determinant for sensitivity to inhibition of Aurora Kinase A (AURKA).^40^ Interestingly, cell lines with high MYC expression were sensitive to AURKA inhibition, while those with low expression of MYC were not.^40^ This is due to the AURKA inhibitor, MLN8237, promoting degradation of MYC through disruption of MYC/AURKA complexes, leading to cell death in cells dependent on high levels of MYC for survival.^40^ MYC can also be stabilized by AURKB to prevent its degradation by the proteosome.^46^ Specifically, AURKB phosphorylates MYC to promote its stability, and MYC in turn directly activates AURKB transcription.^46^ It has been further shown in T-cell acute lymphoblastic leukemia that inhibition of AURKB using AURKBi leads to tumor suppression *in vivo*.^46^ Future studies should therefore compare the role of MYC in response to AURKB inhibition in advanced thyroid cancer.

A major limitation of AURK inhibitors is their toxicity.^47^ As shown in Supplementary Figure S4, CDK1, CDK2 and PLK1 were other top hits in the KEA, all of which are highly involved in cell cycle progression and likely contribute to toxicity. While there are many small molecule inhibitors of CDK1 and CDK2, none have reached FDA approval.^48^ There currently exists one ATP-competitive inhibitor of PLK1 approved for treatment of AML,^49^ and there is evidence to suggest the FDA approved proteosome inhibitor bortezomib and the approved anti-alcohol abuse drug Disulfiram both inhibit expression of PLK1.^50,51^ These and other PLK1 inhibitors currently in clinical trials should be considered in future studies for treatment of cancers with high AURK activity. In support of this, in differentiated thyroid cancers, treatment with the pan-PLK inhibitor volasertib and the multi-kinase inhibitor sorafenib enhanced growth inhibition *in vitro* and *in vivo*.^52^ Thus, these alternate targets represent other strategies to target the AURK signaling axis.

Future studies should explore how inhibition of ERK1/2 and AURKB prevent cell growth and induce apoptosis. ERK1/2 has many cytoplasmic and nuclear targets that promote growth and prevent apoptosis, including the pro-apoptotic protein, MCL1.^53^ Further, members of the ETS transcription factor family, including ELK1, EGR1, and ETS1/2, are ERK1/2 targets that are capable of binding to the promoter of *AURKB*, according to ChIP-Enrichment Analysis (CHEA).^54^ These data indicate that direct inhibition of ERK1/2 may also result in a downregulation of AURKB gene expression. Accordingly, our previous RNA sequencing data show that inhibition of ERK1/2 with ulixertinib reduces AURKB expression, while BRAF inhibition alone does not.^55^ Finally, AURKB not only plays an important role in chromosome segregation and cell division, but also has many substrates involved in apoptosis, including TP53.^56^ Together, these data suggest that combined AURKB and ERK1/2 inhibition slows cell growth and promotes apoptosis in *BRAF-*mutant advanced thyroid cancer cells and represents a promising therapeutic strategy for patients who do not respond to current therapies.

## Financial support

1R01CA222299 (RES), 1F31CA257079 (HMH), 1F31CA247211 (MMR), T32CA174648 (MMR), and University of Colorado Cancer Center support grant P30CA046934.

## Supplementary Material

SupplementaryFigures_revision_FINAL.docx

## Data Availability

The data generated in this study are available upon request.
