# Supplementary material for: The effects of Aurora Kinase inhibition on thyroid cancer growth and sensitivity to MAPK-directed therapies
Source: Cancer Biol Ther. 2024 Mar 23;25(1):2332000. doi: 10.1080/15384047.2024.2332000 (PMC10962586; doi:10.1080/15384047.2024.2332000)
Supplement: SupplementaryFigures_revision_FINAL.docx [file KCBT_A_2332000_SM3551.docx]

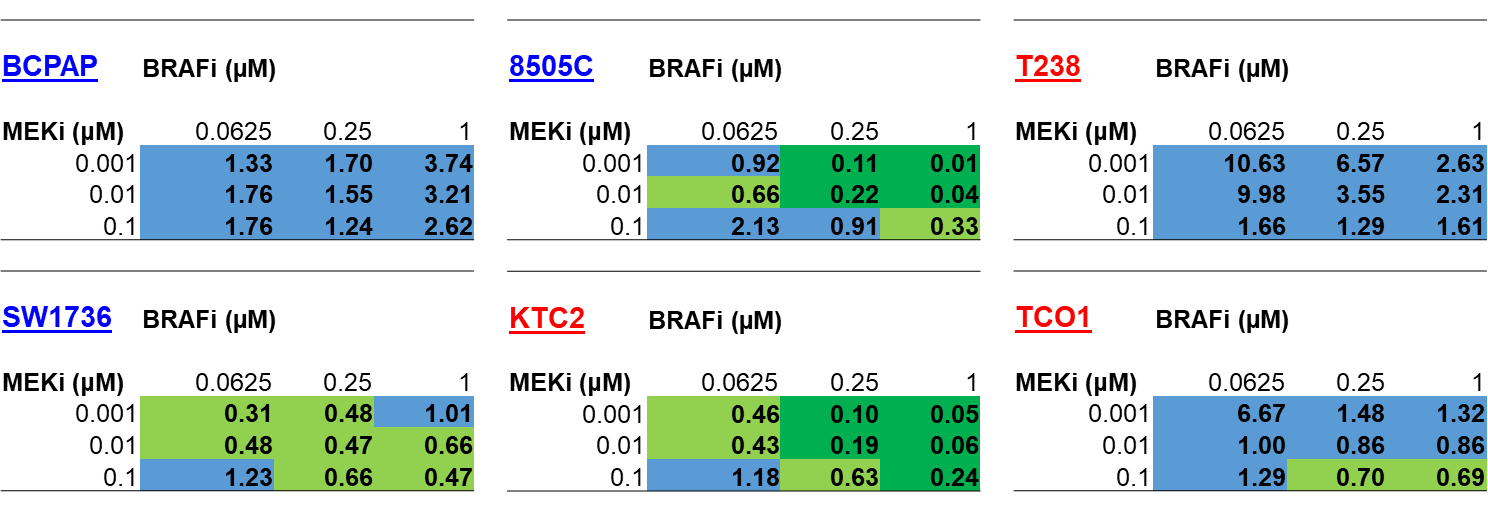


Supplementary Figure 1 – Combined BRAF and MEK1/2 inhibition has synergistic effects on cell growth. Individual CI values corresponding to cell viability from Figure 3.2 are shown. Strong synergy, Combination Index (CI) < 0.3 is shown in dark green, Moderate synergy CI 0.3 - 0.75 is shown in light green, and blue indicates mild synergy or less (CI > 0.75). MEKi-sensitive cell lines shown in blue, MEKi-resistant cell lines shown in red. BRAFi: dabrafenib; MEKi: trametinib.


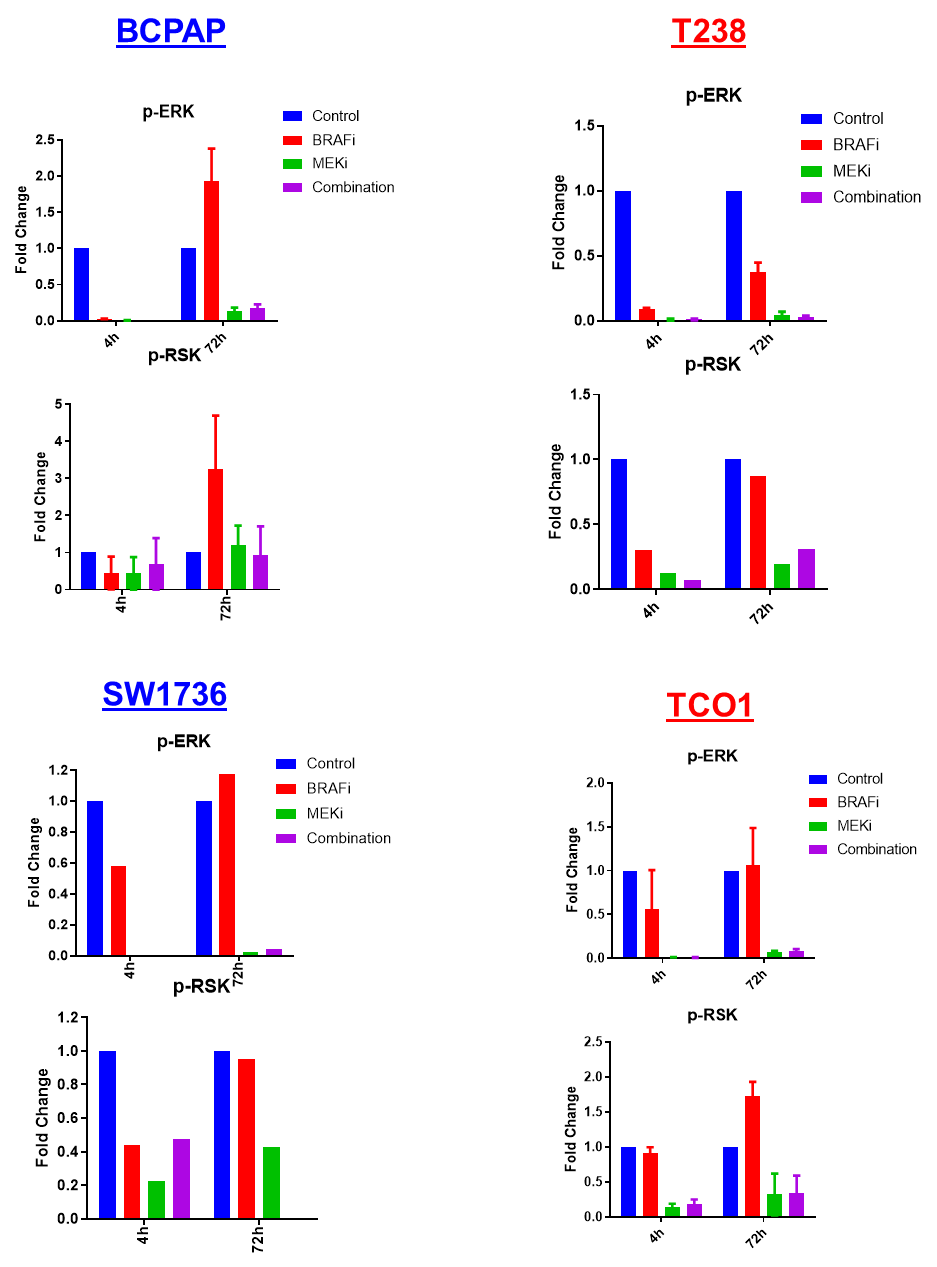


Supplementary Figure 2. Quantitation of Combined BRAF and MEK1/2 inhibition. The Licor Odyssey was used for protein quantification of the Western blots in Figure 2. Proteins were normalized to loading control.

**
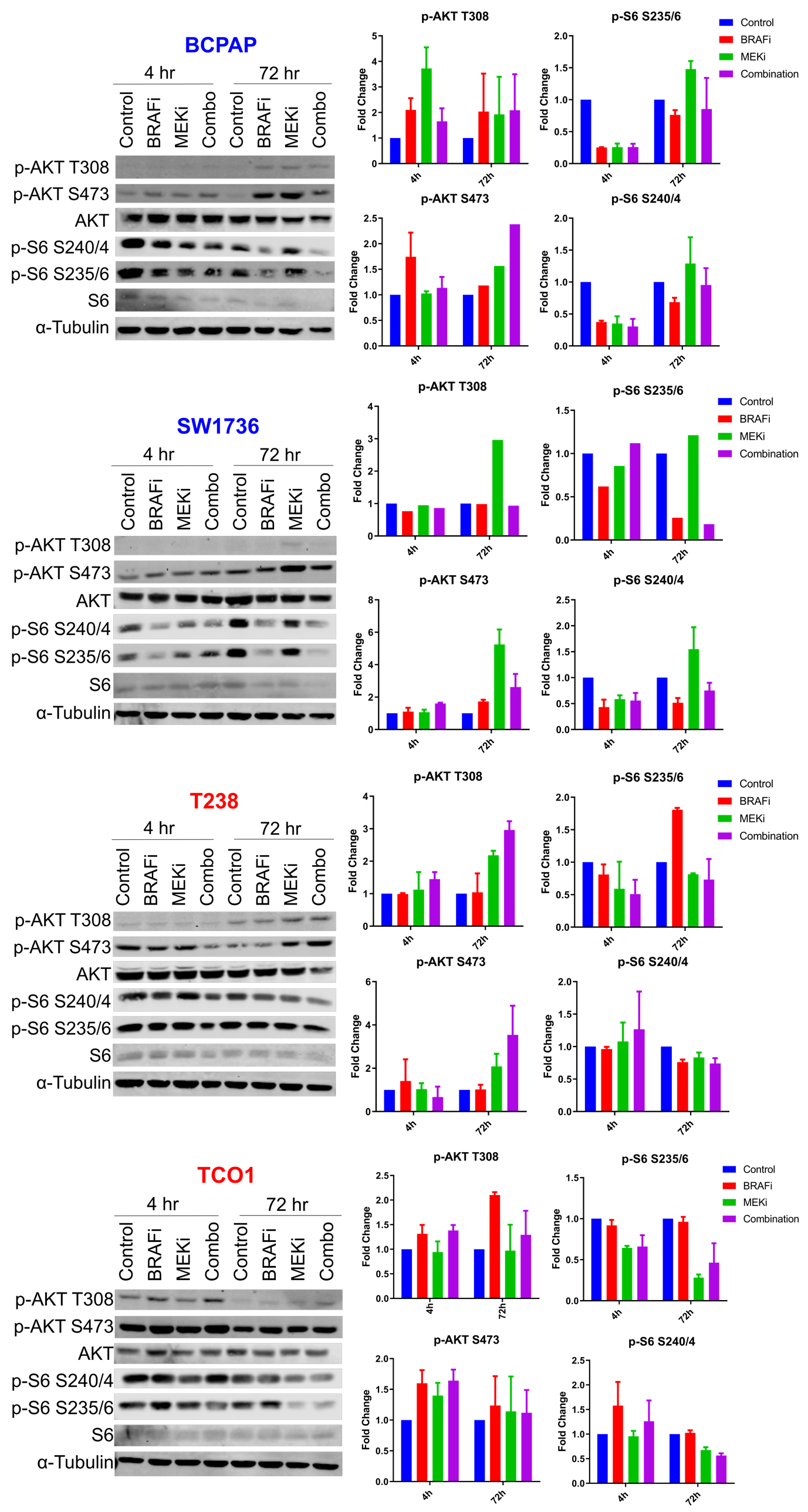
**

Supplementary Figure 3 – The effect of combined BRAF/MEK1/2 inhibition on the PI3K/AKT/mTOR pathway. Four cell lines (SW1736, BCPAP, T238, and TCO1) were treated with BRAFi, MEKi, or both for 72 hrs then re-challenged with drug or combination for 4 hrs prior to sample collection. Lysates were then collected and analyzed by immunoblot with the antibodies shown. Quantification of fold-change in protein levels normalized to loading control. Cell lines in blue are MEKi-sensitive. Cell lines in red are MEKi-resistant. Data as mean fold change +/- SEM from two individual experiments. BRAFi: 50 nM dabrafenib; MEKi: 100 nM trametinib.


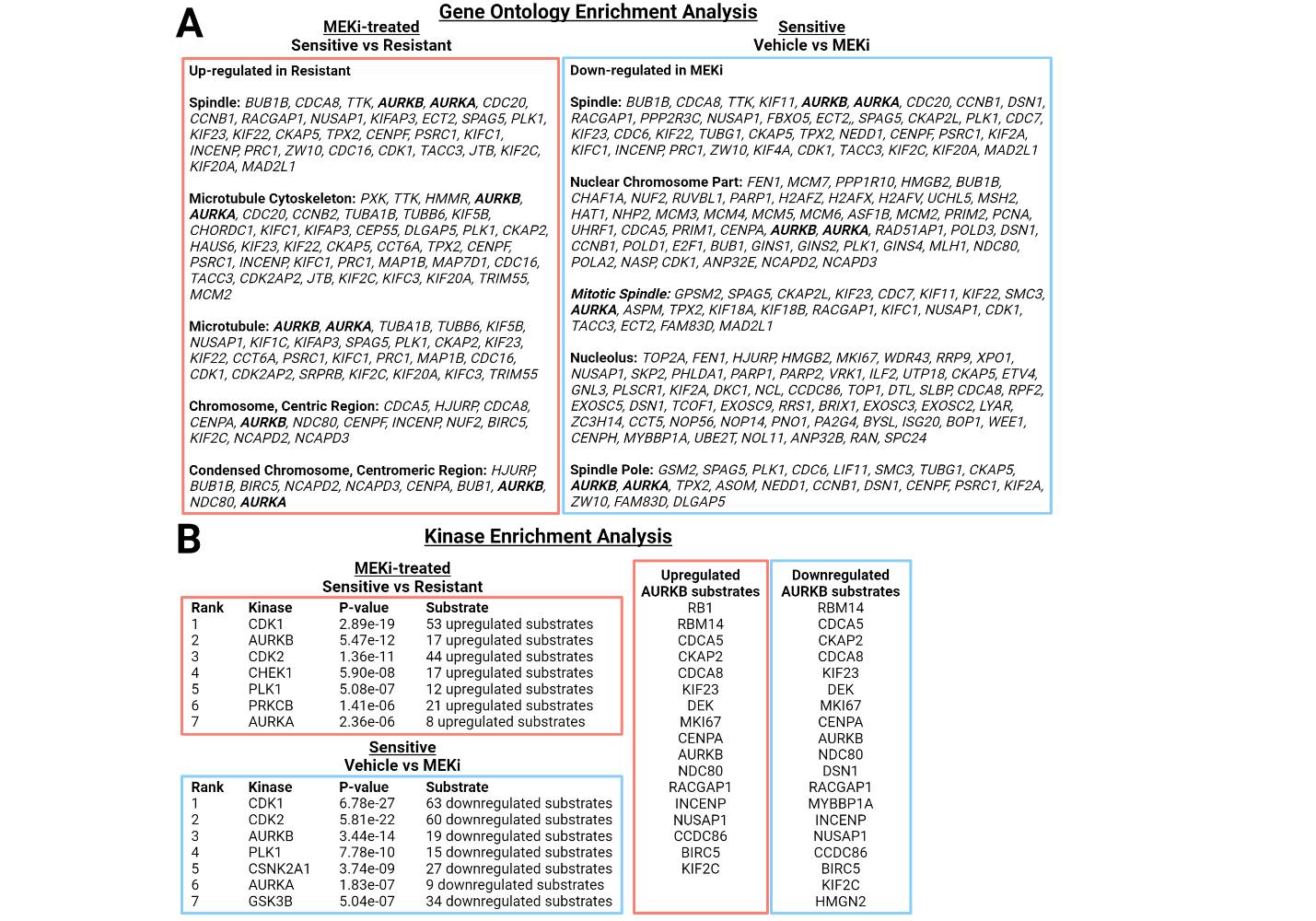


Supplementary Figure 4 – AURKB expression and substrates are downregulated in sensitive cell lines in response to MEKi. RNA-seq through the University of Colorado Cancer Center Shared Genomics Core was performed on two MEKi-sensitive (BCPAP, 8505C) and two MEKi-resistant (T238, TCO1) *BRAF*-mutant cell lines treated with vehicle or MEKi for 48 hrs. BioJupies^37^ was used to perform gene ontology analysis and kinase enrichment analysis, summarized in Figure 3.4. **A)** Genes in the top five regulated gene ontology terms. **B)** Kinase enrichment analysis results and respective upregulated and downregulated substrates of AURKB are shown. MEKi: 100 nM trametinib.


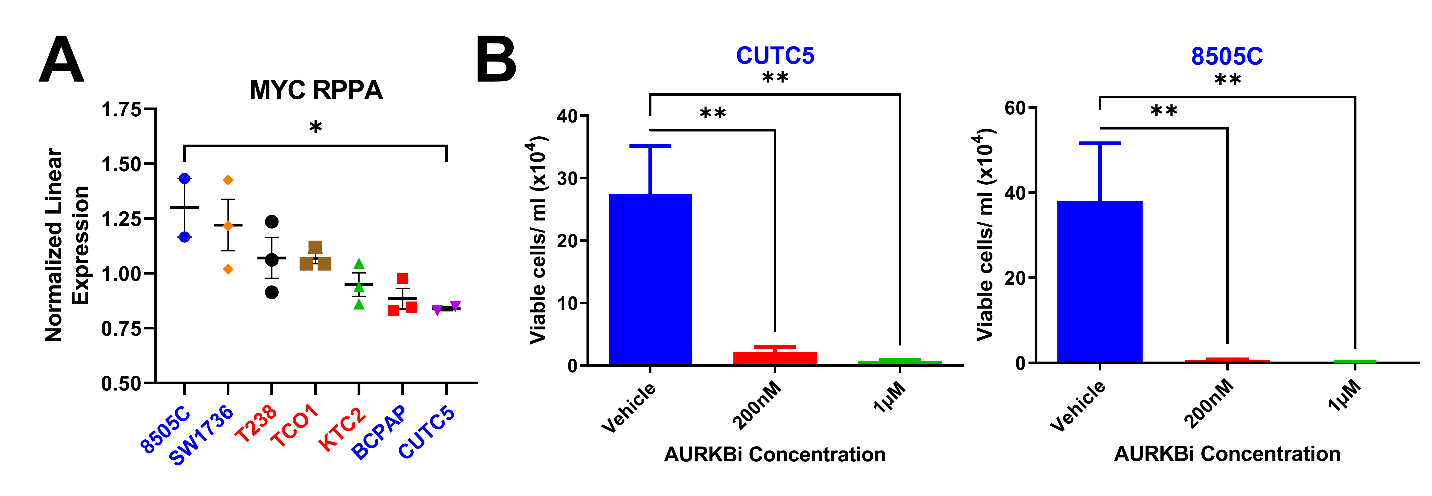


Supplementary Figure 5 – Expression of MYC does not predict sensitivity to AURKB inhibition in *BRAF-*mutant thyroid cancer. A) Protein expression of MYC was quantified in seven cell lines using RPPA (MD Anderson Functional Proteomics Reverse Phase Protein Array Core). MEKi-sensitive cells shown in blue, MEKi-resistant cells shown in red. B) *BRAF*-mutant thyroid cancer cell lines with low (CUTC5) or high (8505C) MYC expression were treated with barasertib (AURKBi). After 72 hrs, viable cell count was determined using a Vi-Cell Cell Viability Analyzer. Results displayed as mean +/- SEM of three experiments. *, p-value <0.05; **, p-value < 0.01. AURKBi: barasertib.
